# Efficacy of middle meningeal artery embolization in the treatment of refractory chronic subdural hematoma

**DOI:** 10.4103/2152-7806.73801

**Published:** 2010-12-13

**Authors:** Masaki Mino, Shinjitsu Nishimura, Emiko Hori, Misaki Kohama, Shingo Yonezawa, Hiroshi Midorikawa, Mitsuomi Kaimori, Teruhiko Tanaka, Michiaharu Nishijima

**Affiliations:** Department of Neurosurgery, Aomori Prefectural Central Hospital, 2-1-1 Higashitsukurimichi, Aomori 030-8553, Japan; 1 Department of Radiology, Aomori Prefectural Central Hospital, 2-1-1 Higashitsukurimichi, Aomori 030-8553, Japan; 2 Department of Clinical Laboratory, Aomori Prefectural Central Hospital, 2-1-1 Higashitsukurimichi, Aomori 030-8553, Japan

**Keywords:** Chronic subdural hematoma, embolization, middle meningeal artery, recurrence

## Abstract

**Background:**

There are no established treatment procedures for repeatedly recurring chronic subdural hematoma (CSH). In this study, we discussed the efficacy of middle meningeal artery (MMA) embolization in preventing recurrence of CSH.

**Methods:**

We performed superselective angiography of MMA in four patients who suffered from repeated recurrence of CSH. After angiography, we performed embolization of MMA with endovascular procedure.

**Results:**

In all cases, superselective angiography of MMA revealed diffuse abnormal vascular stains that seemed to represent the macrocapillaries in the outer membrane of CSH. In all the patients, there were no recurrences or enlargements of CSH after the embolization of the MMA.

**Conclusion:**

MMA embolization can be an effective adjuvant procedure in preventing the recurrence of CSH.

## INTRODUCTION

Single-burr-hole surgery (closed-system drainage or irrigation with or without drainage) has been established as an effective procedure for the treatment of chronic subdural hematoma (CSH).[3,11] However, in several patients, CSH recurs repeatedly, and there are no established treatment procedures for such cases. In this study, we have discussed the efficacy of middle meningeal artery (MMA) embolization in the prevention of CSH recurrence.

## MATERIALS AND METHODS

Between January 2007 and December 2008, 75 patients with CSH underwent single-burr-hole surgery, closed-system drainage, or irrigation of the hematoma cavity. Among these patients, four patients (5.3%) showed repeated recurrences of CSH or progressive reaccumulation of the fluid in a short period.

**Figure 1:**
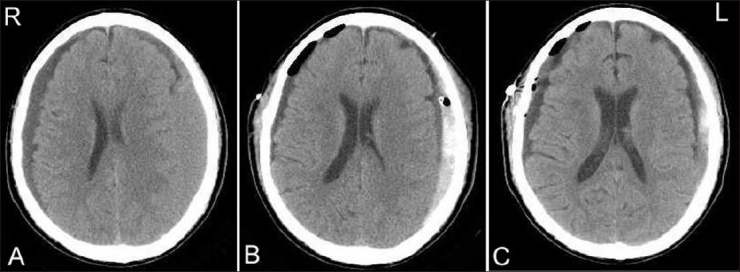
CT images of case 1. (A) CT scan on admission showing bilateral CSH. (B) CT scan 3 months after first surgery, showing recurrence of left CSH. (C) CT scan 2 months after second surgery, showing recurrence of left CSH

The first patient (case 1) was a 73-year-old man who presented with gait disturbance. He had suffered a fall accident 1 month before admission. The computed tomography (CT) images revealed bilateral CSH [Figure 1A]. The patient underwent bilateral closed-system drainage after burr-hole craniotomy. The patient’s gait disturbance resolved immediately after surgery. However, 3 months later, he developed right hemiparesis, and CT scan revealed recurrence of left CSH [Figure 1B]. The patient underwent irrigation of the recurrent CSH, but within 2 months after the surgery, the hematoma showed a gradual increase in size [Figure 1C].

**Figure 2:**
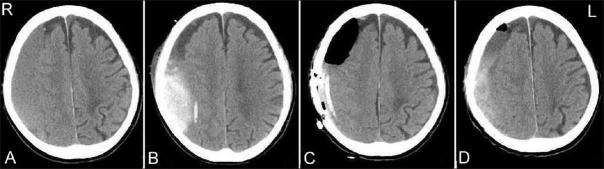
CT images of case 2. (A) CT scan on admission showing right CSH. (B) CT scan 1 day after the surgery, showing a bleeding between the dura mater and the outer membrane. (C) CT scan after the craniotomy. (D) CT scan 2 weeks after the craniotomy, showing an increase in right subdural hematoma

The second patient (case 2) was a 79-year-old man without any history of trauma. The patient presented with speech disturbance and dementia, and CT scan revealed right CSH [Figure 2A]. The patient underwent closed-system drainage. However, the day after the surgery, the patient suffered from bleeding between the dura mater and the outer membrane of CSH [Figure 2B], and he underwent evacuation of hematoma with craniotomy [Figure 2C]. The outer membrane was removed and the hemiparesis was resolved, but the subdural hematoma increased gradually even after the craniotomy [Figure 2D].

**Figure 3:**
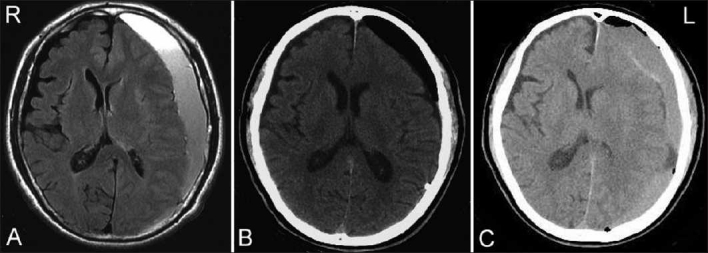
MRI and CT images of case 3. (A) MRI on admission showing left CSH. (B) CT scan after irrigation and drainage. (C) CT scan 13 days after the surgery, showing recurrence of left CSH

The third patient (case 3) was a 65-year-old man who presented with right hemiparesis. He had suffered a fall accident 1 month before admission. The magnetic resonance images revealed left CSH [Figure 3A], and the patient underwent irrigation of the hematoma after single-burr-hole craniotomy [Figure 3B]. The surgery was completed uneventfully; however, by 13 days after the surgery, the hematoma had recurred and increased to its previous size [Figure 3C].

**Figure 4:**
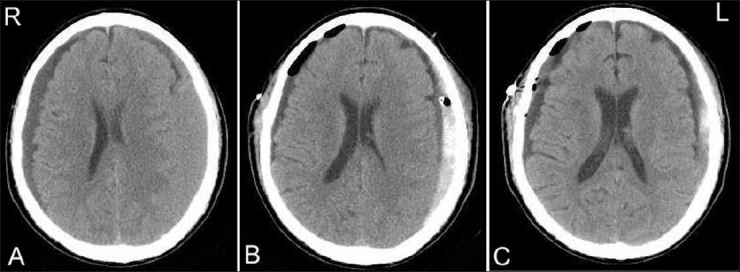
CT images of case 4. (A) CT scan on admission, showing bilateral CSH. (B) CT scan 1 day after the surgery, showing a new hemorrhage in the left side of hematoma cavity. (C) CT scan 2 weeks after the surgery, showing an increase in left subdural hematoma

The fourth patient (case 4) was a 75-year-old man who presented with gait disturbance. He had been involved in a motorcycle accident 1 month before admission. The CT image revealed bilateral CSH [Figure 4A], and the patient underwent bilateral closed-system drainage. The surgery was completed uneventfully, but the CT image obtained on the day after surgery revealed a new hemorrhage in the left side of the hematoma cavity [Figure 4B]. The patient was observed conservatively, but the hematoma volume gradually increased [Figure 4C].

In these four cases, superselective angiography was performed using a microcatheter that was inserted into the MMA, ipsilateral to the recurrent CSH. After angiography, MMA was embolized in the endovascular procedure with gelatin sponge and Guglielmi detachable coils.

## RESULTS

Table 1 shows the summary of the four cases. All four patients were men, and their mean age was 73 years (range, 65–79 years). Three patients had a history of head trauma. All the cases were initially treated with single-burr-hole surgery (three patients were treated with closed-system drainage, and one patient was treated with irrigation and drainage), but all the patients showed enlargement of CSH.

**Figure 5:**
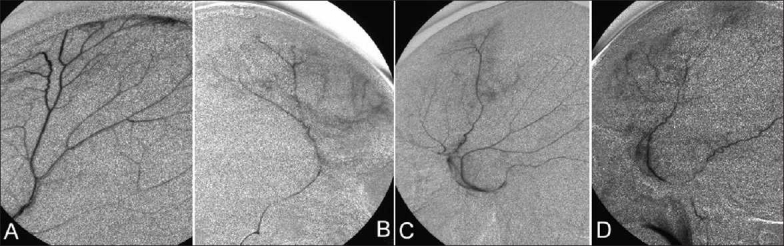
Superselective angiography of MMA (lateral view, ipsilateral side of the recurrent CSH) in cases 1 to 4 (A-D), showing diffuse abnormal vascular stains around the branches of MMA

**Figure 6:**
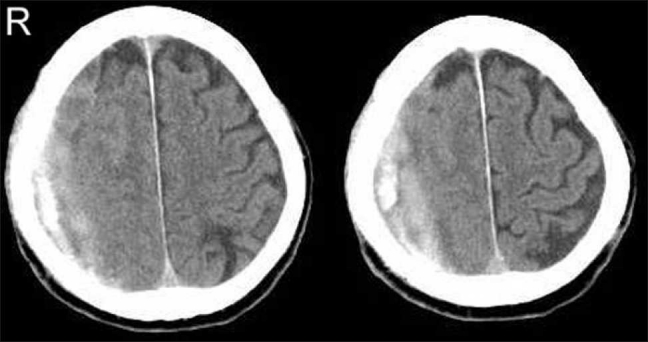
CT image of case 2 obtained after the MMA angiography procedure, showing leakage of contrast enhancement into the hematoma cavity

In all the four cases, superselective angiography of MMA revealed diffuse abnormal vascular stains around the branches of MMA, which seemed to represent the macrocapillaries in the outer membrane of CSH [Figure 5]. After the embolization of MMA, these vascular stains disappeared in the external carotid angiography in all the cases. In case 2, a CT image obtained after angiography revealed leakage of the contrast enhancement into the hematoma cavity [Figure 6].

**Table 1:** Summary of cases

Case No	Age (yrs), Gender	Side	Previous history of trauma	First treatment	Second treatment	Abnormal vessels in DSA	Treatment after embolization of MMA	Recurrence after embolization of MMA
1	73, M	L	Yes	Closed-system drainage	Irrigation with drainage	Yes	Closed-system drainage	No
2	79, M	R	No	Closed-system drainage	Evacuation of hematoma with craniotomy	Yes	Conservative treatment	No
3	65, M	L	Yes	Irrigation with drainage	-	Yes	Closed-system drainage	No
4	75, M	L	Yes	Closed-system drainage	-	Yes	Conservative treatment	No

MMA - middle meningeal artery; DSA - digital subtraction angiography

Two patients (the first and the third patient) underwent further drainage within 24 hours after embolization, and two patients were observed without any surgical treatment. All the patients were observed for more than 6 months after the embolization of MMA, and there was no recurrence or enlargement of CSH. In the two patients who were conservatively observed after embolization, the rest of the CSH gradually reduced.

## DISCUSSION

In the literature, the recurrence rate of CSH has been reported to be approximately 10%.[8,14,11] The recurrence rate seems to be higher in the patients showing coagulopathy or loss of brain volume.[5,10] Several techniques have been used for the management of frequently recurring CSH; these include placement of Ommaya reservoir,[2,10] subdural-peritoneal shunt,[1,7,16] removal of the outer membrane using craniotomy,[15] and perforation of the septum with an endoscope.[9,4] These treatments are occasionally effective, but the procedures are not radical, and some of these procedures are quite invasive. The treatment of recurrent hemorrhagic disorders should focus on addressing the origin of the bleeding.

Tanaka *et al*.[12] performed superselective angiography of MMA in 35 patients with CSH, and they found diffuse dilatation of MMA and scattered abnormal vascular networks, which seemed to represent the macrocapillaries of the outer membrane. In our study, the abnormal vascular stains were observed in the MMA angiography results for all the cases. Tanaka *et al*.[13] also performed a histological study of the vascular structures of the outer membrane of CSH, and they found that there are three types of vessels (capillary-like vessels, small veins, and small arteries) that penetrate through the dura mater and connect to MMA. These studies suggest that the meningeal arteries feed the outer membrane and affect the enlargement of CSH. The contrast enhancement leakage into the hematoma cavity observed in case 2 is consistent with this suggestion. MMA occlusion can stop the blood supply to the outer membrane, thereby preventing the enlargement or recurrence of CSH.

MMA embolization for the treatment of CSH was first reported by Mandai *et al*. in 2000,[6] and this approach prevented the recurrence of CSH in a patient with liver cirrhosis. In the four cases analyzed in our study, the clinical course after embolization of MMA was satisfactory; no patients showed recurrence of CSH, and in two cases, no further treatments were necessary. This result highlights the efficacy of MMA embolization in the prevention of CSH recurrence.

## CONCLUSION

Because CSH is more frequent in elder patients, repeated surgery can be invasive and torturous for the patient. MMA embolization has been established as a less-invasive procedure for the treatment of meningiomas or dural A-V shunts.

Surgery is still the appropriate form of treatment for CSH; however, MMA embolization can be an effective adjuvant procedure to prevent or delay the recurrence of CSH.
